# A Perspective Review on Numerical Simulations of Hemodynamics in Aortic Dissection

**DOI:** 10.1155/2014/652520

**Published:** 2014-02-03

**Authors:** Wan Naimah Wan Ab Naim, Poo Balan Ganesan, Zhonghua Sun, Kok Han Chee, Shahrul Amry Hashim, Einly Lim

**Affiliations:** ^1^Department of Biomedical Engineering, Faculty of Engineering Building, University of Malaya, 50603 Kuala Lumpur, KL, Malaysia; ^2^Department of Mechanical Engineering, Faculty of Engineering Building, University of Malaya, 50603 Kuala Lumpur, KL, Malaysia; ^3^Discipline of Medical Imaging, Department of Imaging and Applied Physics, Curtin University, Perth, WA 6845, Australia; ^4^Department of Medicine, Faculty of Medicine Building, University of Malaya, 50603 Kuala Lumpur, KL, Malaysia; ^5^Department of Surgery, Faculty of Medicine Building, University of Malaya, 50603 Kuala Lumpur, KL, Malaysia

## Abstract

Aortic dissection, characterized by separation of the layers of the aortic wall, poses a significant challenge for clinicians. While type A aortic dissection patients are normally managed using surgical treatment, optimal treatment strategy for type B aortic dissection remains controversial and requires further evaluation. Although aortic diameter measured by CT angiography has been clinically used as a guideline to predict dilation in aortic dissection, hemodynamic parameters (e.g., pressure and wall shear stress), geometrical factors, and composition of the aorta wall are known to substantially affect disease progression. Due to the limitations of cardiac imaging modalities, numerical simulations have been widely used for the prediction of disease progression and therapeutic outcomes, by providing detailed insights into the hemodynamics. This paper presents a comprehensive review of the existing numerical models developed to investigate reasons behind tear initiation and progression, as well as the effectiveness of various treatment strategies, particularly the stent graft treatment.

## 1. Introduction

Aortic dissection is characterized by splitting of the aortic wall caused by tear in the intima layer that allows blood to enter the media layer through an intimomedial entrance tear, subsequently dividing the layer into true and false lumens [1–3]. The dissections may propagate either distally or proximally from the entry tear and may involve vessel branches, leading to malperfusion syndrome [1, 3]. The Stanford system is used by physicians to categorize aortic dissection into type A (the ascending aorta is affected) and type B (ascending aorta is not affected) [1, 2, 4, 5].

Several risk factors have been associated with the development and progression of aortic dissection, including hypertension [3, 6, 7], race and sex [6], genetic factors, connective tissue disease [6, 7], aging, and previous repair of aortic aneurysm or dissection [2]. Among these, hypertension constitutes the highest risk. Chronic exposure to high pressure in the aorta leads to wall thickening, fibrosis, calcification, and extracellular fatty acid deposition [1, 3]. On the other hand, genetic factors such as Marfan's syndrome, vascular Ehlers-Danlos syndrome, bicuspid aortic valve, and familial aortic dissection contribute to a compromised intima and aortic dissection through differentiation of the vascular smooth muscle cells and enhanced elastolysis of the aortic wall components [3].

Aortic dissection may lead to serious complications, including aortic rupture, myocardial ischemia, aortic regurgitation, cardiac tamponade, hypotension/shock, end organ ischemia, and death [2]. Hypotension or shock generally indicates a rupture or impending rupture of the aorta in type B aortic dissection, and this normally occurs in the elderly patients [8]. On the other hand, obstruction of branches arising from the aorta, such as subclavian artery, coronary arteries, and renal arteries, may cause malperfusion syndrome [2], leading to serious consequences such as permanent paraplegia [9]. The occurrence of malperfusion syndrome is less common in the elderly compared with the younger patients due to more localized dissection being reported in the elderly patients [8]. Studies have shown that the effect of dissection on perfusion to a branch artery depends on whether the artery is perfused by the false or true lumen, relative collapse of the true lumen, and relation of the intimal flap to the branch artery [2].

Numerous methods have been used to diagnose aortic dissection in order to devise the best treatment strategies for the patients involved. These involve computed tomography (CT) (61% usage), transoesophageal echocardiography (TEE) (33% usage), aortography (4% usage), and magnetic resonance imaging (2% usage) [6, 10]. CT and TEE are normally used at the initial investigation of suspected acute dissection [2, 4, 11, 12]. CT provides information about the extent of aortic involvement [2, 10, 11], while TEE has the advantages of defining the mechanism of aortic regurgitation, visualizing the coronary ostia, and ascertaining the function of the left and right heart. Apart from the various imaging modalities, biomarkers such as elastin fragments [13], D-dimers [2, 13], and smooth muscle myosin heavy-chain protein have also started to attract wide interest.

Numerical simulation methods, particularly the computational fluid dynamics (CFD) approach, involve study and analysis of blood flow patterns as well as other hemodynamic variables in selected vessel domains. It provides the means through which reproducible numerical experiments can be produced under identical conditions. Over the years, it has emerged as a reliable tool which serves to enhance our understanding of the pathophysiology and progression of aortic disease, as well as a predictive tool for treatment outcomes. Extensive numerical studies have been performed to investigate changes in the flow dynamics in a normal thoracic aorta [14, 15], dissected aorta [7, 16–28], and aortic aneurysm [29, 30], as well as to predict outcomes of stent graft and surgical treatments. Evolving from early CFD techniques using simplified geometries [16, 18, 19, 28, 31–35], more recent studies have utilized patient-specific geometry reconstructed from MRI and CT data [7, 14, 15, 19–27]. In the last decade, fluid-structure interaction (FSI) models that take into account the interaction between the blood and the vessel wall have been developed [29, 31, 33].

The present paper provides an overview of the existing therapeutic management of aortic dissection and their limitations, followed by a comprehensive review of the existing numerical studies of flow in diseased conditions, focusing on their methods, findings (hemodynamic variables), and validations of the results. Special emphasis is placed on factors causing initiation and progression of aortic dissection and effectiveness of the different therapeutic approaches, particularly stent graft treatment.

## 2. Therapeutic Approaches to Aortic Dissection

Medical treatment is currently the preferred method of choice for uncomplicated type B aortic dissection patients [12, 36], particularly in patients with stable hemodynamic status, no branch vessel involvement, and no periaortic hematoma [8, 36]. The primary goals are to prevent the propagation of the false lumen, prevent rupture, accelerate healing, and reduce the risk of complications [11, 37, 38] by lowering aortic wall stress. Three parameters have been reported to affect aortic wall stress, that is, velocity of ventricular contraction, rate of ventricular contraction, and blood pressure [38]. In view of this, beta-blocker therapy and vasodilators, which act to reduce heart rate and blood pressure, have commonly been used [12]. Medical treatment has, however, been associated with frequent incidences of organ malperfusion, as a result of extension of the dissection, expansion of the aneurysm, and compression of the adjacent structures [37].

Surgical intervention is indicated in most patients with ascending aorta (type A) dissections [9], as well as in some patients with descending aortic (type B) dissection who show rapid expansion of a dissecting aneurysm, blood leakage, impending rupture, persistent and uncontrollable pain despite medical therapy, recurrent and/or refractory pain, and/or impairment of blood flow to an organ or limb [8, 36, 39–41]. The goals of the surgical therapy in type A dissection are to stop retrograde extension of the dissection and to prevent rupture of the ascending aorta. In type B the aim is to replace the dissected aorta [11, 37]. To date, mortality rate using surgical management has been reportedly high for both types of dissections [37]. Few predictors of operative mortality have been identified, including the presence of cardiac tamponade, the site of the tear, the time to operation, the presence of renal/visceral ischemia, renal dysfunction, and the presence of pulmonary disease [37, 40].

In view of the complication rate associated with surgical treatment, less invasive percutaneous intervention such as aortic fenestration and stent graft placement has been increasingly used in clinical practice in type B dissection [4]. Percutaneous fenestration, which involves opening of the intimal flap, aims to provide a reentry tear for the dead-end false lumen back into the true lumen [4]. On the other hand, stent grafts facilitate aortic remodeling by covering the proximal primary intimal tear in order to promote false lumen thrombosis and subsequently eliminating flow into the false lumen, at the same time of scaffolding the true lumen [11, 42]. Despite its advantages, stent graft placement poses several complications, including migration associated with ventricular ejection, proper size selection, and endoleaks [43].

## 3. Numerical Simulations in Aortic Dissection

Compared to information provided by state-of-the-art medical imaging diagnostic tools in the context of aortic dissection, the numerical simulation approach further provides hemodynamic variables such as blood flow dynamics, pressure distribution, wall shear stress/rate, mass transport, and recirculation region. As a result, it has been extensively used to investigate pathological flow in the blood vessel and evaluate the efficacy of various treatment strategies. Existing studies that applied numerical simulations in studying aortic dissection are discussed below.

### 3.1. Geometrical Structures

#### 3.1.1. Extraction of Geometries from Imaging Modalities

The standard imaging methods used to obtain realistic aortic geometries from patients with aortic dissection for simulation purposes are computed tomography (CT), magnetic resonance imaging (MRI), and magnetic resonance angiography (MRA). Among these modalities, CT, which provides images with high spatial resolution, is most commonly used. On the contrary, the usage of MRI in these patients is less frequent due to long acquisition time (and therefore poorer accessibility), as well as lower spatial resolution. Nevertheless, it offers an advantage of not requiring ionizing radiation; instead, contrast is achieved by exploiting differences in the magnetic spin relaxation properties of the various bodily tissues and fluids.

#### 3.1.2. Idealized Geometry versus Realistic Geometry

Table 1 shows a summary of settings that have previously been used by researchers in the simulations of aortic dissection. With regard to aortic geometry, both idealized and patient-specific geometries reconstructed from images obtained from CT, MRI, and MRA [16, 21–24, 28] have been used. Simplifications in geometrical structures are commonly made in simulations performed using idealized geometry, including constant lumen diameter from the ascending aorta to the aortic arch [18, 19, 28], straight vessels [19, 28], and the exclusion of branches [16, 19, 28]. In an actual geometry, tapering occurs from the ascending aorta to the descending aorta, causing an increase in the velocity magnitude along the vessel [44, 45]. Furthermore, instead of straight vessels, the aortic geometry is tortuously curved at the descending aorta and bent slightly toward the posterior left side, followed by slight concave curvature toward the anterior aspect [20]. This changes the velocity profile, pressure, and wall shear stress distribution along the vessel [20]. On the other hand, exclusion of branches in the simulation of aortic dissection overestimates the distribution of mass flow rate (MFR) to the true and false lumen at the descending aorta. MFR is important as it is considered as a factor leading to false lumen dilation [20]. Although various studies have attempted to use patient-specific geometries for more realistic simulation, the number of patients used in these studies is very limited, with a maximum number of 12 patients in one study [25].

#### 3.1.3. Tear Configurations

Available idealized or patient-specific geometries have been modified to simulate the effect of different tear scenarios, including the effect of occlusion in the entrance or reentrance tears [21, 22, 28], variations in the false and true lumen area [16, 28], shape and size of the tears [28, 48], and inclusion of the intimal flap between the true and false lumen (Table 2). Two assumptions have been made on the shape of the tears, that is, an elliptical and a circular shape. Generally, it was observed clinically that tears in aortic dissection are closer to an elliptical shape [7, 20]. On the other hand, large variations in the true and false lumen as well as tear diameters have been used in numerical simulations, as shown in Table 2. In actual aortic dissection geometry, there is a separator between the true lumen and the false lumen known as the intimal flap, often with tears along the flap. Most simulations do not include this, but instead a gap was introduced to separate the true and false lumens.

#### 3.1.4. Stent Graft

Several studies [21–23, 25–28] have simulated the treatment of aortic dissection using stent graft. However, instead of using an actual geometry for stent graft design in these simulations, the stent graft treatment has been simulated by simply occluding the entrance tears [22, 28]. Meanwhile, separate simulations have been carried out to evaluate the efficacy of stent graft designs alone, where the stent graft template was either extracted from the contrast enhanced transaxial thoracic CT scan images of patients [25–27] or represented by a smooth, nontapering, no out of plane bending circular tube.

### 3.2. Material Properties

#### 3.2.1. Rigid Wall versus Fluid-Structure Interaction Study

FSI simulation has been implemented in several studies related to blood vessels, including a simple axisymmetric three-layered wall model of a descending aorta [33] and another study on normal aorta [31]. However, to date, we are not aware of any FSI studies on aortic dissection. Instead, all numerical studies have assumed the wall and the intimal flap to be rigid [7, 20, 47] (Table 1). In an actual clinical scenario, the intimal flap was observed to contract (3.5 ± 0.9 mm) and extend (3.0 ± 2 mm), in a pattern correlated with the difference between the true and false lumen pressure [47]. Further investigation to determine the properties of the vessel wall and intimal flap would be beneficial, particularly in simulating stent graft treatment, as the device is directly hooked to the flap. The main difficulty in performing a FSI study for aortic dissection is that the vessel wall contains both healthy and diseased tissue, for which the exact material properties are difficult to be ascertained.

In an attempt to more closely simulate the movement of the vessel wall, Midulla et al. [24] incorporated prescribed wall movement obtained from cine MRI in their numerical studies. Movement of the vessel wall, together with flow and pressure measurements obtained from various imaging modalities, could serve as an important source of data in estimating the wall properties.

#### 3.2.2. Property of the Blood

Most numerical studies on aortic dissection have assumed the blood to be Newtonian (Table 1), based on several factors: (i) negligible effect of particles in a large artery [16, 25, 27, 28]; (ii) high shear rates in the large arteries [7]; (iii) to reduce computational workload [31]. Although both Gao et al. [31] and Cheng et al. [20] showed that non-Newtonian model causes minor differences in the basic flow characteristics, wall shear stress and pressure distribution, Cheng et al. [20] revealed that non-Newtonian model caused a reduction in the maximum WSS value (~8%) and pressure magnitude (~12%). Furthermore, the inclusion of non-Newtonian model has either extended or reduced the turbulence intensity at different regions along the vessel.

In another study, Hou et al. [18] investigated the effect of modeling the blood as a multiphase medium that consists of plasma (61% and 55%), red blood cells (RBC) (38% and 44%), and leukocytes (1%). Only RBC was modeled as non-Newtonian in their study, as it controls the blood rheology [49]. As suggested by Jung et al. [49], the usage of multiphase flow, which gives better insight into the interaction between the particulate and the biomechanical factors, could further explain the causative link between biomechanical factors and arterial pathogenesis.

### 3.3. Flow Settings

#### 3.3.1. Extraction of Flow and Wall Movement from Imaging Modalities

Although aortic geometries are normally extracted from CT, information about flow and wall movement is not available from this modality. In order to measure these two parameters, phase contract MRI (PCMRI) and cine MRI are used, respectively [24]. Both flow and wall movements are important in providing realistic boundary conditions for the simulations as well as to serve as sources of validation data for simulation results.

#### 3.3.2. Laminar, Transitional versus Turbulent Flow

Most of the studies [7, 25–27] assumed blood flow to be laminar, based on the justification that, in a large vessel, mean flow velocity is low and therefore Reynolds number is relatively low [7, 28, 46] (Table 1). Furthermore, it has been well established that flow can be assumed to be laminar if the maximum Reynolds number along the geometry is less than the critical Reynolds number (based on the Womersley number). Using this method, several studies on aortic dissection [7, 25–27] have justified their flow to be laminar.

**Figure 1:**
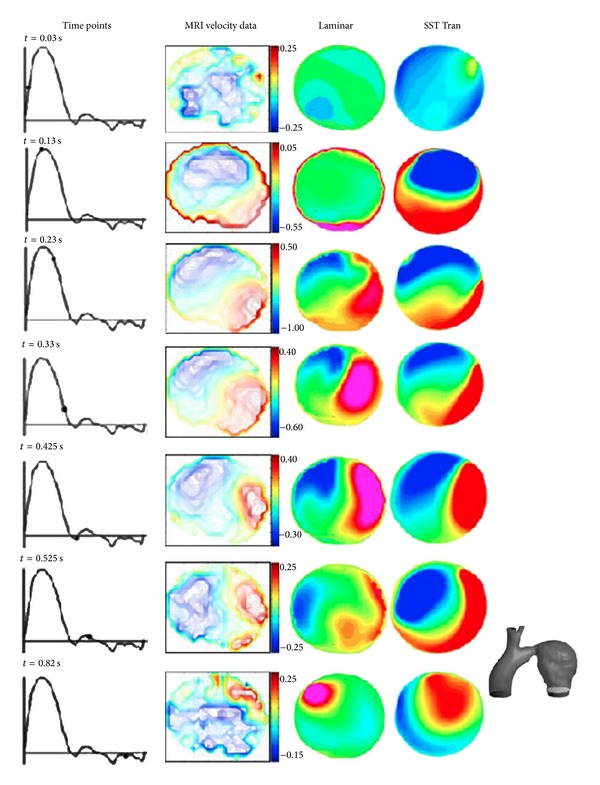
Comparison of axial velocity profiles at different time points along the cardiac cycle between MR velocity data (in m/s) and simulation results using (i) laminar flow model and (ii) SST transitional flow model (SST Tran) [30].

A study by Tan et al. [30] on thoracic aneurysm demonstrated that transitional model could reproduce the velocity contour obtained from MRI more accurately as compared to using laminar flow model (Figure 1). Based on this finding, Cheng et al. [20] and Karmonik et al. [47] assumed the flow to be transitional in their simulation study and used the correlation based transitional version of Menter's hybrid k-e/k-w SST Tran model for their simulations on aortic dissection. On the other hand, Khanafer and Berguer [33] and Hou et al. [18] assumed the flow to be turbulent in their studies.

### 3.4. Applications of Numerical Simulations in Aortic Dissection

To date, numerical simulations have been used to predict progression of the disease, particularly dilation of the aorta [7, 16, 20, 46, 47] and formation of thrombus [18, 28], and effect of various treatment strategies, including thromboexclusion, fenestration, and stent graft [21–23, 28, 48], as well as to investigate biomechanical factors leading to failure of stent graft devices [25–27].

#### 3.4.1. Tear Initiation in Aortic Dissection

**Figure 2:**
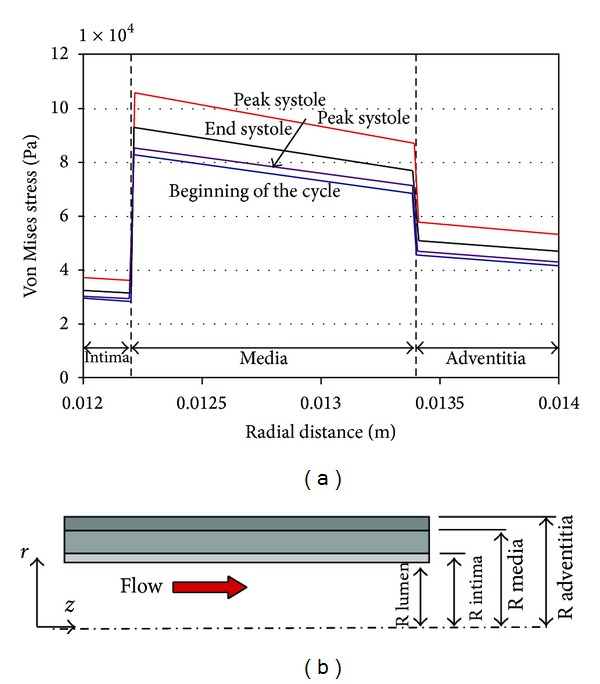
The highest Von Mises stress across the wall of a descending aorta at various periods of the cycle was found at the medial layer as shown in (a) whereas (b) shows the axisymmetric idealized three-layered model of a descending aorta. Adapted from Khanafer and Berguer [33].

In a FSI study by Khanafer and Berguer [33], they found that the medial layer, which has the largest elasticity, showed the highest wall stress and shear stress as shown in Figure 2. Turbulent and Newtonian blood flow settings were used, with different thickness ratios (13/56/31) and elasticity (2/6/4 MPa) for the aortic walls. Results showed that the differences in the elastic properties among the different layers may be associated with the occurrence of dissection in the media layer. In a similar numerical study by Gao et al. using a 3D layered aortic arch model [31], it was reported that the circumferential wall stress is directly related to systolic blood pressure, and this explains the fact that 70–90% of patients with aortic dissection have high blood pressure. Furthermore, similar to Khanafer's study, they demonstrated a higher stress in the media layer as compared to the intima and adventitia layers.

Wen et al. [14] performed both numerical simulations and in vitro experiments on thoracic aorta to investigate the correlation of wall shear stress, pressure, and oscillatory wall shear stress index with aortic disorders, particularly aortic dissection. The geometry of a normal human thoracic aorta for the purpose of in vitro experiments was obtained using the Phase-Contrast MRI through rapid prototyping, while validation of numerical simulations was performed using measurements obtained from the Phase-Contrast MR Velocimetry. They revealed that the maximum wall shear or wall pressure coincides with the initial location of thoracic aorta dissections and subsequently hypothesized that type A dissection which originates in the ascending aorta is most likely the result of both higher values of wall shear stress and wall pressure in the aortic arch, while type B dissection tends to be caused by higher wall shear stress.

Using a different approach, Beller et al. [50] investigated the correlation between aortic root displacement and tear progression in aortic dissections using a finite element model of the human aortic root, aortic arch, and supra-aortic vessels. Displacement and twist were applied to the aortic root base, while 2 different luminal pressures were simulated to represent control and hypertension conditions. Aortic root displacement and high blood pressure have been shown to cause a significant effect on the longitudinal stress in the ascending aorta, which may explain the frequent occurrence of circumferential intimal tears and aortic dissections in this location.

#### 3.4.2. Progression of Aortic Dissection

Numerical simulation, able to provide physical flow conditions through reproducible numerical experiments, plays an important part in predicting the likelihood of disease progression through identifying potential morphological and/or biomechanical factors [20]. Accurate prediction of disease progression, for example, whether imminent rupture or dilation would occur, has a substantial clinical impact as it helps to determine the timing of surgical/intervention treatment for a patient [16]. In a clinical study performed across 101 patients with type B acute dissection without complications, Marui et al. [51] reported that a maximum aortic diameter of >40 mm and a patent false lumen during the acute phase serve as important predictors for aortic enlargement in the chronic phase. Therefore, these patients should undergo surgery before enlargement of the aorta, while those with a maximum aortic diameter of <40 mm should continue with hypotensive therapy. Tang et al. [16] did a CFD study using a 3D aortic model reconstructed from CT images of a patient with thoracic aortic dissection to investigate the effect of six biomechanical factors, that is, (i) size of the dissecting aneurysm, (ii) blood pressure, (iii) geometry around the distal tear, (iv) partial thrombosis, (v) distance between the tears, and (vi) shear stress on force on the walls of the false lumen in order to predict chances of rupture. Their results were in line with the clinical findings by Marui et al. [51], which showed that an increase in the size of the dissection as well as blood pressure increases force acting on the false lumen wall. In a latest simulation study by Karmonik et al. [47], the importance of primary entry tear size and position in affecting the amount of blood flowing into the false lumen and therefore disease progression has again been highlighted.

**Figure 3:**
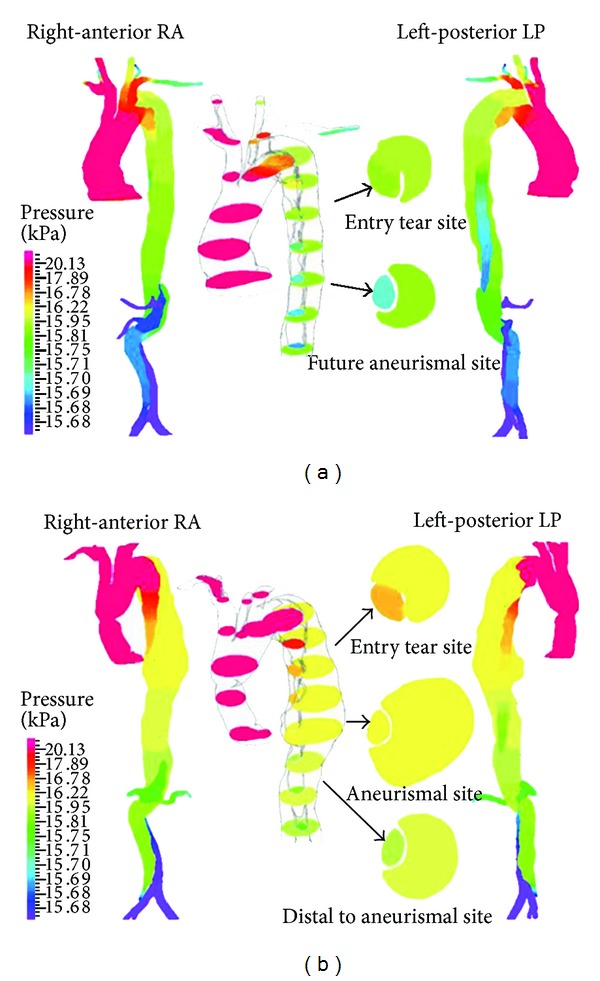
Pressure contour plots adapted from Tse et al. [7]. (a) The preaneurismal aorta (before formation of the luminal aneurysm); (b) the postaneurismal aorta (after formation of luminal aneurysm). In the middle, an interior view of random axial cross-sections shows pressure difference in true and false lumen.

Although aortic diameter has been clinically used as a guideline to predict aortic dissection dilation, it is believed that hemodynamic parameters (e.g., pressure and wall shear stress), geometrical factors, and mechanical properties of the aorta wall played a key role in disease progression [7]. Patency of the false lumen and partial thrombosis and prolonged high wall shear stress have been listed as markers on the prediction of dilation [19]. In a CFD study using patient-specific dissecting aneurismal aortas before and after the formation of luminal aneurysm, Tse et al. [7] found that high pressure difference between the true and false lumens in the preaneurismal aorta coincides with the false luminal aneurysm in the descending aorta as shown in Figure 3. Furthermore, a region of elevated time-averaged wall shear stress was found at the entry tear and is believed to cause the extension of tear. Apart from that, Tse et al. [7] inferred from their results that the wrapping of the false lumen around the true lumen may be associated with the helical nature of hemodynamic flow in the human aorta.

The highly disturbed flow pattern in type B aortic dissection was likely to induce turbulent zones, and this led to the use of transitional, turbulence flow model by Cheng et al. [20] in their numerical simulation. Results showed that the dissected aorta was dominated by highly disturbed (involving helical flows) and possibly turbulent flow with strong recirculation within both the false and true lumens, in particular in the region surrounding the tear. High levels of time-averaged wall shear stress were found at the coarctation throat and the edge of the tear, increasing the likelihood of tear expansion and reduction of aortic distensibility. Around the region near the tear, high turbulence intensity values were found. Although both laminar and turbulent flow simulations produce qualitatively similar distribution of wall shear stress, significantly higher magnitude was obtained with the transitional turbulence model.

In another study, Karmonik et al. [17] employed CFD simulations to identify hemodynamic changes associated with disease progression, using patient-derived geometries from a 3D contrast enhanced MRI study at the initial examination and a CT angiography study at 10-month follow-up. Using geometrical data from the patient, they were able to reproduce the hemodynamic changes caused by false lumen dilatation. It was shown that false lumen dilatation occurring during the period between initial and follow-up study led to lower blood flow velocities at the false lumen, thus reducing total pressure and overall wall shear stress.

Considering the importance of hematocrit content on viscosity and fluid dynamic behavior of the blood, Hou et al. [18] attempted to investigate the thrombosis mechanisms in aortic dissection. It was demonstrated from their results that the red blood cell has a tendency to separate from the plasma and coagulate at the bottom of the false lumen, leading to high viscosity in this region, which may induce the formation of thrombus.

#### 3.4.3. Therapeutic Management of Aortic Dissection

Owing to its minimally invasive procedure which reduces the mortality rate and speeds the recovery time, stent graft placement has become one of the major treatment strategies for type B dissection. In view of this, numerical simulations aimed to investigate the efficacy of stent graft treatment and evaluate various stent graft designs have attracted wide interest.

Karmonik et al. [22, 23] performed a series of simulation studies on the effect of endovascular stent graft placement in aortic dissection. Computational fluid dynamics were performed with aortic geometry derived from MR angiographic images and inflow conditions (i.e., aortic flow waveform) measured with 2D phase contrast MRI. The entrance tear was virtually occluded to simulate endovascular stent graft treatment, by eliminating inflow into the false lumen. Their results agreed with that reported by Tsai et al. [52], who found a decrease in both systolic and diastolic false lumen pressure with entry occlusion, despite a low variability for true lumen systolic pressures. One of the interesting results in their study was the occurrence of a reversal in the pressure difference between true and false lumens at end systole. They further extended their study to quantify wall shear stress and dynamic pressure changes after endovascular treatment with dynamic MRI measurements obtained from a patient pre- and post-(1 week) stent graft placement, as well as at 1-year follow-up. It was observed that the large wall shear stress and dynamic pressure found at the entrance tear and a stenotic region in the true lumen were significantly reduced, that is, by more than a factor of 2, after the endovascular graft treatment. Only a small posterior section of the false lumen remained after the treatment, and the antegrade flow within the lumen was eliminated. Two regions of elevated dynamic pressure were found within the stent graft, which may lead to stent graft failure.

Although endovascular treatment has proven to be a promising strategy, issues still remain on the effect of the degree of thrombosis (or, in other terms, the level of false lumen patency) on potential rupture of the false lumen postoperatively and whether a secondary procedure should be undertaken. Fan et al. [28] employed CFD to assess the effect of 3 features, that is, (i) the ratio of the area of the false lumen to that of the true lumen, (ii) the size of the reentry tear, and (iii) the position of the reentry tear, on the extent of thrombosis in the false lumen after endovascular stent graft treatment. Increasing the area ratio of the true lumen to the false lumen was found to lower the risk of false lumen rupture, as it creates a larger domain of stagnant fluid in the false lumen. On the other hand, patients with a larger reentry tear are at a higher risk, as this increases blood flow motion into the false lumen through the reentry tear. Lastly, the position of the reentry tear along the descending aorta has a negligible effect on the formation of thrombosis.

**Figure 4:**
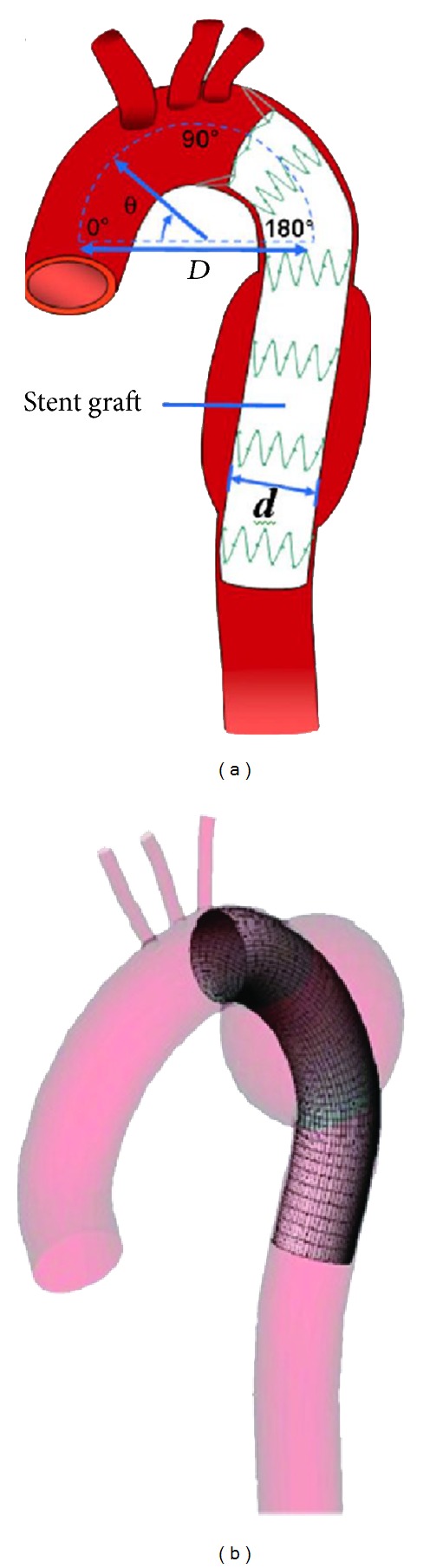
(a) The picture shows the factors affecting the drag force on stent graft which are the diameter of curvature, *D*, the internal diameter of stent graft, *d*, and the phase angle of curvature or the starting position of the graft, **θ**. (b) Mesh elements are created for the fluid domain. Adapted from [27].

Apart from studies aiming to investigate the effect of endovascular stent graft placement, several researches have also looked into the effect of various biomechanical factors on stent graft remodeling and migration using numerical simulations. As reported by Lam et al. [26], an inappropriate proximal landing zone, a lack of a healthy distal attachment site, and minimal oversizing could led to stent graft instability in the long term. Three factors affecting the drag force on a stent graft were analyzed using CFD techniques [25, 26], that is, (i) the internal diameter of the stent graft, (ii) the starting position of the graft, and (iii) the diameter of curvature of the aortic arch as shown in Figure 4. In their simulation results, drag force increases linearly with increasing in the internal diameter, due to an increase in the mass flow rate. On the other hand, the drag force decreases considerably with the starting position shifting downwards, that is, from the proximal end to more distal end of the aortic arch. Flow separation and secondary flows occur at the aortic arch due to the effect of curvature, leading to substantial energy dissipation. To the contrary, diameter of curvature of the aortic arch plays a less significant effect on drag force, thus posing minimal risk of migration to the stent grafts.

**Figure 5:**
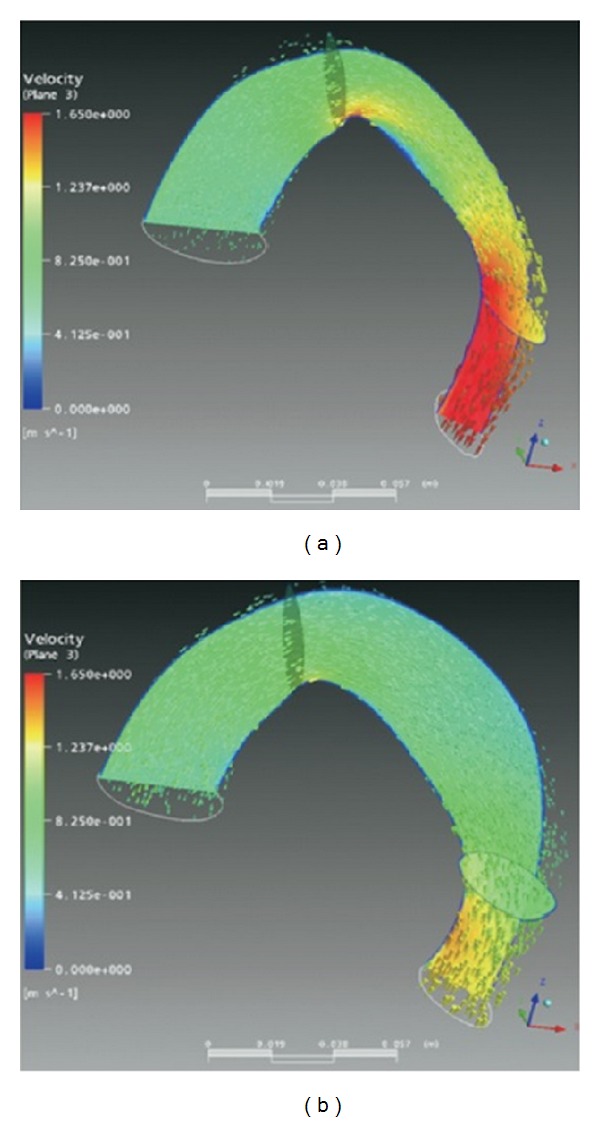
Model of fluid dynamics velocity and vector plot in a thoracic stent graft before (a) and after (b) remodeling. Adapted from Cheng et al. [25].

In a subsequent study, Cheng et al. [25] followed up 12 patients with type B aortic dissections implanted with thoracic stent graft for more than 12 months. Serial CT scans of patients revealed that there was a general increase in both inlet and outlet graft areas after endovascular stent graft treatment as in Figure 5. Furthermore, substantial remodeling of the stent graft was observed in these patients. The increase in the area of the stent graft is associated with an increase in the resultant drag force on the stent graft. Apart from geometrical factors, the same research group also extended their study to investigate the effect of dynamic factors [27], that is, blood pressure level and waveform, as well as blood viscosity on drag force. It was shown from their results that increasing blood pressure level and systolic slope of the pressure waveform substantially increased the drag force, which contributes to stent graft failure. On the other hand, blood viscosity had a milder effect on drag force.

Apart from endovascular stent graft therapy, fenestration and thromboexclusion are alternative surgical treatment strategies for aortic dissection. Using patient-specific geometry derived from MR images, Karmonik et al. [23] performed a simulation study examining the effect of fenestration on flow patterns and pressures in aortic dissection by completely removing the intra arterial septum. Surgical fenestration was found to reduce the systolic pressure from 2400 to 800 Pa in the combined lumen. Using a slightly different approach, Wan Ab Naim et al. [48] studied the impact of additional reentry tears (more than 2) on the flow, pressure, and wall shear stress distribution in the dissected aorta. With an increasing number of reentry tear, pressure was reduced, which was believed to prevent false lumen expansion and reduce the possibility of true lumen collapse. As reported by Panneton et al. [53], surgical fenestration helped to relief organ or limb ischemia.

Surgical thromboexclusion involves bypassing the dissected aorta and creating flow reversal in the dissected segment [41, 54]. Researchers from Beijing University of Technology [55–57] performed numerical simulation on idealized 2D geometric models for type B aortic dissection to investigate the effect of bypass graft on pressure and flow patterns. Bypass graft operation has been shown to reduce pressure and blood flow activities in the false lumen, and this is believed to alleviate the propagation of aortic dissection and promote thromboexclusion in the dissecting channel.

## 4. Future Directions

Numerical simulation greatly contributes to diagnosis and surgical planning by providing clinicians and surgeons with a better insight into the disease through revealing various hemodynamic factors that are difficult to be measured in vivo. However, due to different limitations, these simulations are always done with underlying assumptions which may affect the accuracy of the simulation results. As such, result validation is of paramount importance, where matched outcomes between simulated results and findings from in vitro experiments or imaging modalities are desired. To have compatible results, the boundary conditions as well as the flow assumption (laminar/transitional/turbulent) should be as realistic as possible.

With the advancement of imaging technologies, patient-specific models have been increasingly popular. To increase robustness of the model, numerical simulations involving a large number of patient-specific geometries with various configurations should be performed, taking into consideration the complexity of the aortic dissection geometries and the huge variability across patients. Apart from that, follow-up imaging studies on patients with aortic dissection who went through surgical treatments would be useful to validate numerical simulation results aimed to predict disease progression and treatment outcomes. These models can then be reliably applied for treatment planning purpose.

On the other hand, CFD studies have progressively stepped forward to FSI which provides a more realistic flow analysis with vessel wall mechanics interaction. Movement of the interarterial septum, which may contribute to changes in blood flow in the true and false lumen, should be taken into account. One major obstacle in FSI models of aortic dissection is the determination of the material properties of different wall layers as well as the different material properties between a healthy and diseased tissue, which can only be obtained accurately through isolated experiments. One could take advantage of the recent advancements in parameter optimization, where material properties could be obtained through various sources of data.

To date, no studies have looked into the interaction between aortic dissection and stent graft designs. A potential problematic spot for stent graft placement is at the point of the distal arch because the device is unable to conform to such abruptly angled geometry [58]. In order to determine the best location for stent graft placement which minimizes endoleak, the actual stent graft geometry should be incorporated with realistic geometry of aortic dissection. For this particular purpose, advanced methods of FSI with contact surface methodology could be implemented.

## 5. Summary and Conclusion

A comprehensive overview on the applications of numerical simulations in aortic dissection has been presented, focusing on biomechanical factors leading to tear initiation and progression, as well as prediction of treatment outcomes. Both idealized geometry and patient-specific models have been developed, with the help of various medical imaging modalities. Vessel wall properties and complicated geometrical and biomechanical factors are found to be closely correlated with tear initiation and progression. High levels of blood pressure and time-averaged WSS significantly increase the likelihood of false lumen rupture. With various surgical treatments such as endovascular stent graft placement, bypassing the dissected aorta, and fenestration, WSS and dynamic pressure will be substantially reduced.

## Figures and Tables

**Table 1:** Summary of the numerical simulation settings for aortic dissection.

Number	Imaging modalities	Geometrical parameters	Number of patients	Flow	Blood property	Wall	Multiphase/single phase	Reference
Realistic geometry								
1	CT and MRA	—	2	Laminar	Newtonian	Rigid	Single phase	[46]
2	CT	—	4	Transitional flow	Newtonian	Rigid	Single phase	[47]
3	CT	Inlet diameter (ascending aorta) = 36.0 mm	1	Transitional flow	Newtonian and non-Newtonian (Quemada model)	Rigid	Single phase	[20]
4	CT	—	2	Laminar	Newtonian	Rigid	Single phase	[7]
5	CT	—	1	Laminar	Newtonian	Rigid	Single phase	[27]
6	CT	—	12	Laminar	Newtonian	Rigid	Rigid	[25]
7	CT	—	1	Laminar	Newtonian	Rigid	Rigid	[26]
8	MRI	—	1	Laminar	Newtonian	Rigid	Single phase	[21]
9	MRI	—	1	Laminar	Newtonian	Rigid	Single phase	[23]
10	MRI	—	1	Laminar	Newtonian	Rigid	Single phase	[22]
11	MRI	—	1	Laminar	Newtonian	Rigid	Single phase	[17]
12	MRA	—	6			Prescribed wall movement		[24]
13	CT	—	1	Laminar	Newtonian	FSI	Single phase	[48]

Ideal geometry								
14	—	Inlet diameter (ascending aorta) = 30.0 mm Curvature diameter of the aortic arch = 93.0 mm Intimal flap thickness = 2 mm	—	Laminar	Newtonian fluid	Rigid	Single phase	[28]
15	—	Inlet diameter (ascending aorta) = 30.0 mm Curvature diameter of the aortic arch = 120.0 mm	—	Turbulent: k-epsilon	Newtonian fluid	Rigid	Single phase	[16]
16	—	Inlet diameter (ascending aorta) = 20.0 mm Intimal flap thickness = 2 mm	—	Laminar	Newtonian fluid	Rigid	Single phase	[19]
17	—	Inlet diameter (ascending aorta) = 25.0 mm	—	Turbulent: SST k-w	Non-Newtonian: RBC Newtonian: plasma Newtonian: leukocyte	Rigid	Three-phase flow	[18]
18	—	—	—	—	Newtonian	FSI	Single phase	[31]
19	—	—	—	Turbulent: SST k-w	Newtonian	FSI	Single phase	[33]

**Table 2:** Tear configurations used in numerical simulations for aortic dissection.

Number	Tear shape	Tear diameter (mm)	True lumen diameter (mm)	False lumen diameter (mm)	Number of tears	Reference
1	Elliptical	Long axis: 22–26 Short axis: 10–15	7.0–12.5	16.5–21.0	1 (reentry tear)	[28]
2	Nearly circular	37	48	62	1 (entry tear)	[20]
3	Spherical	10	20	20	2 (1 entry, 1 reentry)	[19]
4	Elliptical	Long axis: 13 Short axis: 10	40–80	40–80	2 (1 entry, 1 reentry)	[16]
5	Elliptical	Long axis: 6 Short axis: 3	—	—	1 (entry tear)	[18]
6	Elliptical	Long axis: 9–11 Short axis: 16–23	—	—	1 (entry tear)	[7]
7	Elliptical	Long axis: 18–33 Short axis: 9–36	—	—	1 (entry tear)	[47]
